# The complete chloroplast genome of Chinese endemic species *Abies ferreana* (Pinaceae) and its phylogenetic analysis

**DOI:** 10.1080/23802359.2022.2097028

**Published:** 2022-07-12

**Authors:** Dan Wang, Qing-Yin Zeng, Xue-Min Han

**Affiliations:** State Key Laboratory of Tree Genetics and Breeding, Chinese Academy of Forestry, Beijing, China

**Keywords:** *Abies ferreana*, chloroplast genome, phylogenetic analysis

## Abstract

*Abies ferreana* Bordères & Gaussen 1947 is endemic to China, where it is distributed at 3300–4000 meters in the mountains of Southwest Sichuan and Northwest Yunnan. In this study, the complete chloroplast genome of *A. ferreana* was reconstructed by *de novo* assembly using whole-genome sequencing data. The complete chloroplast genome of *A. ferreana* was 120,049 bp in length with a GC content of 37.9%. A total of 113 genes were identified, including 4 rRNA genes, 35 tRNA genes, and 74 protein-coding genes. Among these, 14 genes contain introns. In the phylogenetic tree with 12 other species of *Abies*, *A. ferreana* and *Abies fanjingshanensis* W. L. Huang et al. 1984 were grouped into the same branch, with a bootstrap value of 100%. The complete chloroplast genome of *A. ferreana* provides potential genetic resources for further *Abies* evolutionary and genomic studies.

*Abies* species, commonly known as fir, are a genus comprised of 48–55 species of coniferous evergreen trees in the Pinaceae family (Liu 1971). *Abies* is distributed mainly in the mountains and less frequently on the plains of the northern hemisphere, where they have important ecological and economic value. However, many species of *Abies* have been listed as endangered species (Xiang et al. 2015). Therefore, the classification and conservation of *Abies* has mutual importance for both the ecological and industrial communities. *Abies ferreana* Bordères & Gaussen 1947 is in the second largest genus of Pinaceae and endemic to China. It is found in the mountainous regions of northwest Yunnan and southwest Sichuan at altitudes of 3300–4000 meters. However, current projections indicate a decline in the suitable habitats for *Abies* (Naudiyal et al. 2021). Plant chloroplasts have conserved genomic characteristics and high evolution rates, and are widely used in genetic and evolutionary studies (Rogalski et al. 2015; Yang et al. 2021). We have assembled and characterized the complete chloroplast of *A. ferreana*, which will provide genetic resources for further conservation and evolutionary studies of *A. ferreana*.

This article is licensed under a Regulations of Yunnan Province on biodiversity protection and approved by the Chinese Academy of Forestry (Beijing, China). The sample of *A. ferreana* was collected from Baimaxueshan National Nature Reserve (Yunnan, China; 28°22′57.59″N, 99°0′6.76″E). The total DNA was extracted from fresh leaves for whole-genome sequencing on an Illumina Hiseq-PE150 platform. The DNA and specimen were deposited at the State Key Laboratory of Tree Genetics and Breeding (Website: http://skltgb.caf.ac.cn/, Contact: XM HAN, Email: 602405029@qq.com) under the voucher number TGBZH201010016. A total of 2.84 GB as 150 bp paired-end raw reads were retrieved and then quality-trimmed clean reads were used for the chloroplast genome *de novo* assembly using GetOrganelle with default parameters (Jin et al. 2020). The chloroplast genome of *A. ferreana* was annotated with the online programs, CPGAVAS2 and GeSeq (Michael et al. 2017; Shi et al. 2019; Shi et al. 2019 ). The annotated genomic sequence was registered with GenBank using the accession number OM321039.

The chloroplast genome of *A. ferreana* (OM321039.1) has a total length of 120,049 bp and comprises a small, single-copy (SSC) region of 41,347 bp, a large single-copy (LSC) region of 76,340 bp, and a pair of inverted repeat (IR) regions of 1181 bp each. It encodes 113 genes, including 74 protein-coding genes, 4 ribosomal RNA genes, and 35 transfer RNA genes. Among these genes, 14 genes contain introns, including 6 tRNA (trnK-UUU, trnV-UAC, trnG-GCC, trnL-UAA, trnI-GAU, and trnA-UGC) and 8 protein-coding genes (*rpoC1*, *atpF*, *petB*, *petD*, *rpl16*, *rpl2*, *ycf1*, and *ycf3*). One tRNA gene (*trnS-GCU*) and two protein-coding genes (*psaM* and *ycf12*) were duplicated and located on the IR regions. The overall GC content of the chloroplast genome was 37.9%, and the IR regions (37.8%) had higher GC content than the SSC (37.1%), but lower GC content than the LSC regions (39.0%).

To evaluate the phylogenetic position of *A. ferreana,* 14 complete chloroplast genomes were downloaded from GenBank, including 12 *Abies* and two other gymnosperm species, *Picea abies* (Linnaeus) H. Karsten 1881 and *Pseudotsuga sinensis* var. *wilsoniana* (Hayata) L. K. Fu & Nan Li 1997. A maximum likelihood (ML) tree was constructed with *P. abies* (HF937082.1) and *P. sinensis* var. *wilsoniana* (NC _016986.1) as outgroups using IQ-TREE (Nguyen et al. 2015). The phylogenetic tree showed that *A. ferreana* and *Abies fanjingshanensis* W. L. Huang et al. 1984 were grouped together with a 100% bootstrap support (Figure 1). The complete chloroplast genome of *A. ferreana* fills an important gap in current chloroplast genome information for *Abies* genus of Pinaceae family, providing useful perspectives for further evolutionary and genomic studies in *Abies*.

**Figure 1.**
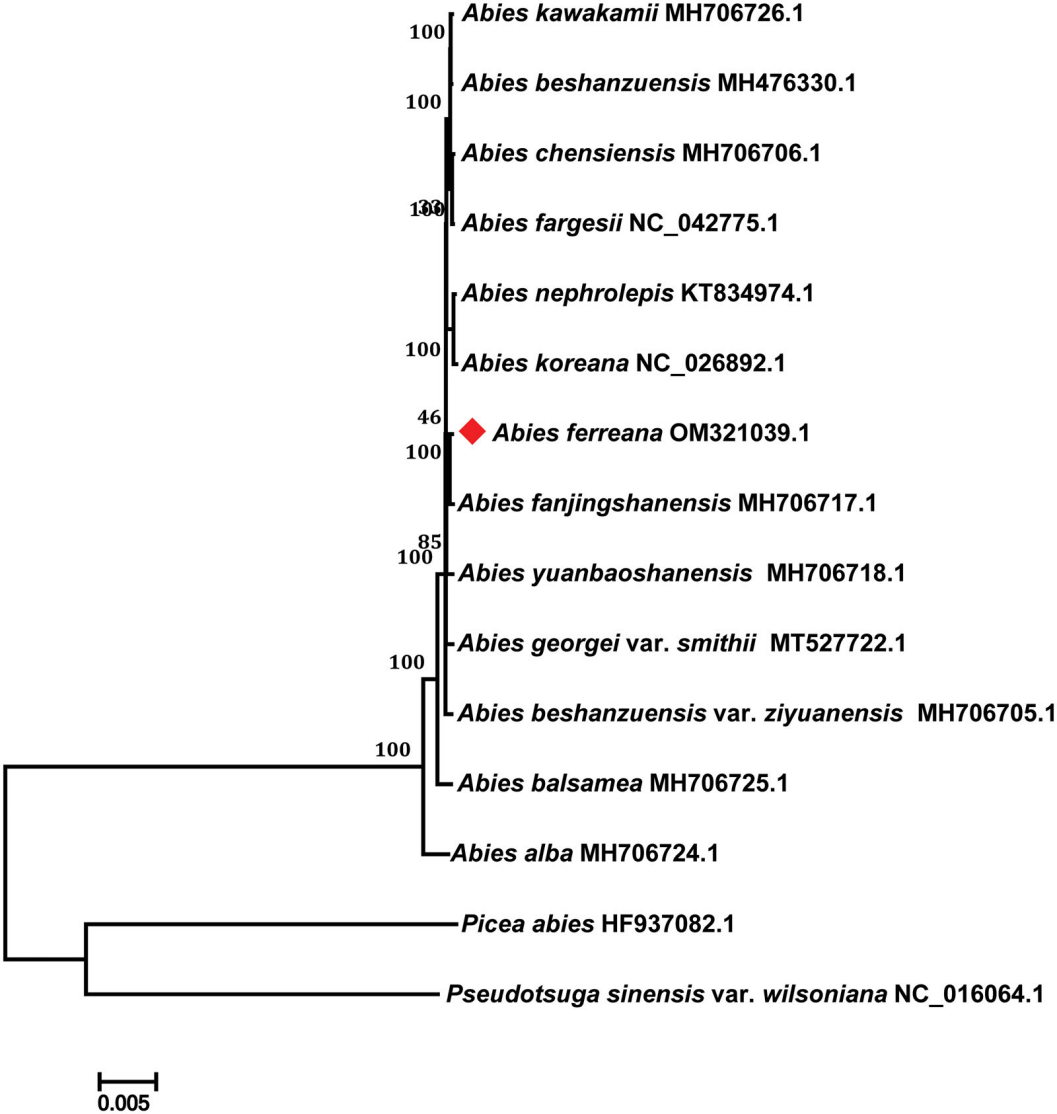
Phylogenetic tree of *Abies ferreana* and other 14 related species based on complete chloroplast genome sequences. Bootstrap values are indicated on the branches.

## Data Availability

The complete plastid genome of *A. ferreana* of this study is available in GenBank with the accession code OM321039.1 (https://www.ncbi.nlm.nih.gov/nuccore/OM321039). Raw data had been uploaded to SRA (https://submit.ncbi.nlm.nih.gov/subs/sra/SUB11339250/overview).The associated BioProject, SRA, and BioSample numbers are PRJNA827276, SRR18779683, and SAMN27608758, respectively.
